# A New Covalent Organic Framework of Dicyandiamide-Benzaldehyde Nanocatalytic Amplification SERS/RRS Aptamer Assay for Ultratrace Oxytetracycline with the Nanogold Indicator Reaction of Polyethylene Glycol 600

**DOI:** 10.3390/bios11110458

**Published:** 2021-11-16

**Authors:** Aihui Liang, Shengfu Zhi, Qiwen Liu, Chongning Li, Zhiliang Jiang

**Affiliations:** 1 Key Laboratory of Ecology of Rare and Endangered Species and Environmental Protection (Guangxi Normal University), Ministry of Education, Guilin 541004, China; ahliang2008@163.com (A.L.); shengfuzhi2020@163.com (S.Z.); qwliu2021@163.com (Q.L.); lcn7882342@163.com (C.L.); 2 Guangxi Key Laboratory of Environmental Pollution Control Theory and Technology, Guilin 541004, China

**Keywords:** COF catalysis, nanogold indicator reaction, aptamer, oxytetracycline, RRS, SERS

## Abstract

In this paper, dicyandiamide (Dd) and p-benzaldehyde (Bd) were heated at 180 °C for 3 h to prepare a new type of stable covalent organic framework (COF) DdBd nanosol with high catalysis. It was characterized by molecular spectroscopy and electron microscopy. The study found that DdBd had a strong catalytic effect on the new indicator reaction of polyethylene glycol 600 (PEG600)-chloroauric acid to form gold nanoparticles (AuNPs). AuNPs have strong resonance Rayleigh scattering (RRS) activity, and in the presence of Victoria Blue B (VBB) molecular probes, they also have a strong surface-enhanced Raman scattering (SERS) effect. Combined with a highly selective oxytetracycline (OTC) aptamer (Apt) reaction, new dual-mode scattering SERS/RRS methods were developed to quantitatively analyze ultratrace OTC. The linear range of RRS is 3.00 × 10^−3^ –6.00 × 10^−2^ nmol/L, the detection limit is 1.1 × 10^−3^ nmol/L, the linear range of SERS is 3.00 × 10^−3^–7.00 × 10^−2^ nmol/L, and the detection limit is 9.0 × 10^−4^ nmol/L. Using the SERS method to analyze OTC in soil samples, the relative standard deviation is 1.35–4.78%, and the recovery rate is 94.3–104.9%.

## 1. Introduction

A covalent organic framework (COF) is a new type of porous conjugated crystalline polymer material. Its framework is mainly composed of C, N, O, and H elements, with strong covalent bond connection, low density, and good chemical stability. In addition, it also has the advantages of large specific surface area, regular pore structure, simple functional design, and easy functionalization; in some cases, it has the characteristics of luminescence and has been applied to analytical chemistry [1]. In analytical methods for COFs, the fluorescence method is interesting due to two-dimensional COFs with rigid delocalization and large π-bond structure with intrinsic luminescence properties [2,3]. Recently, COF catalytic amplification was utilized in a luminescence analysis. Wang et al. prepared a loaded Pd nanocluster COF (BzBD_Pd_) that can catalyze a Au@NiP nanoreaction, combined with aptamer reactions to establish resonance Rayleigh scattering (RRS) methods for the detection of melamine [4]. Therefore, aptamers combined with COFs to establish a new analysis method can be further studied.

Aptamers (Apts) are the main specific oligonucleotides including single-stranded DNA or RNA for varieties of targets obtained through in vitro selection. Under the action of intermolecular forces, for instance, van der Waals forces, hydrogen bonds, hydrophobic, and electrostatic interactions, Apt reactions usually fold into complex and stable three-dimensional structures. Compared to conventional molecular biology cognitive elements, for instance antibodies and enzymes, Apt has considerable merits, including stability in a wider temperature range and pH range, ease of synthesis and modification, lower production costs, and long shelf life [5]. It has been utilized in molecular spectroscopy such as resonance Rayleigh scattering (RRS) and electrochemical analysis, particularly in combination with nanomaterials [6,7]. Li et al. modified AuNPs with anti-epidermal growth factor receptor (EGFR) antibody (Ab) and epidermal growth factor Apt to obtain a multifunctional Apt-AuNP-Ab nano-binding RRS probe [8], which specifically binds to EGFR to form a large-volume binding product, thereby determining EGFR in a functional nanogold system. This method’s linear range is 20–100 ng/mL, and the limit of detection is 0.1 ng/mL. Pourreza et al. utilized AuNPs synthesized in situ to be embedded in polyethanol ether borax hydrogel (PBH) [9], and developed an RRS aptasensor for detecting thrombin from 0.70 pmol/L–0.02 μmol/L with a limit of detection of 0.10 pmol/L.

Surface-enhanced Raman scattering (SERS) is a sensitive molecular spectral technique. Generally, the nanostructured substrates functionalize with Raman reporter molecules, and strong characteristic peaks are generated in the SERS spectrum, thus achieving quantitative detection of the analyte [10]. SERS substrates usually include gold, silver, and other noble metal materials, and the signal intensity of SERS can be effectively enhanced by improving the substrate [11]. Compared with a silver nanostructure, a gold nanostructure has better chemical robustness and perfect functionalized chemistry, and can be better used in SERS substrates [12]. Wang et al. utilized fullerene as the precursor to prepare Au-doped carbon dots (CDAu) under microwave conditions [13]. Through research, it can be concluded that CDAu intensely catalyzes the HAuCl_4_-fructose reaction to produce AuNPs as bifunctional materials of SERS substrates and indicators. On the basis of this, a new method was developed to determine As^3+^ by SERS with a linear range of 0.10–0.60 μg/L and a detection limit of 0.06 μg/L. The combination of the two scatterings with Apt controlled nanocatalytic amplification has made great progress, and has the advantages of both. Li et al. coupled a sensitive carbon dot catalytic multiplicative reaction and a high-selectivity Apt reaction to develop a SERS-RRS scattering spectral technology to determine 5–150 nmol/L potassium ions [14].

Oxytetracycline (OTC) is a compound in the tetracycline class of drugs. It is a broad-spectrum antibiotic and is broadly utilized for veterinary purposes in the livestock and poultry industry. Owing to its low absorption efficiency, any intake above 70% is excreted into the environment through excrement. The non-point-source emissions of municipal waste and the farmyard manure application release antibiotics into the surroundings. They enter natural surface water through urban sewage and wild outflows [15]. The concentration in surface water can be detected in tens of micrograms/liters, and the detection frequency is quite high, up to 90%. The increase in the utilization of OTC and its release into rivers and oceans through wastewater has resulted in OTC resistance genes increasing in pathogen-related species [16]. Therefore, it is urgent to establish new analytical methods for tracing OTC besides classical methods such as chromatography. Ma et al. established a fluorescent Na-Ln heterometallic CP as a fluorescent probe for the quantitative detection of OTC [17]. The linear range was 0–100 μmol/L, and the detection limit was 0.09 μmol/L. Gonzalez et al. proposed an AgNP substrate SERS method to control honey quality and to identify oxytetracycline in honey samples [18]. The detection limit was 0.0001 nmol/mL. Wang et al. prepared silica microspheres coated with CdTe quantum dots to detect 0–80 μmol/L OTC [19]. Yang et al. covalently immobilized the Apt with amino groups on the electrode surface through a diazo coupling reaction [20], and constructed a suitable electrochemical Apt sensing platform for 1.0 × 10^−9^–1.0 × 10^−4^ g/mL OTC. Some of the above methods are not very sensitive, and some of the analysis processes are complicated. As far as we know, there are no reports of a polycondensation reaction of dicyandiamide (Dd) containing C≡N bonds with p-benzaldehyde (Bd) to synthesize the COF of dicyandiamide/p-benzaldehyde (DdBd) with high catalytic activity, and a polyethylene glycol 600 (PEG600)-HAuCl_4_ nanocatalytic amplification indicator reaction, coupled with a highly selective aptamer, to establish a double scattering assay for OTC. In this study, the specific Apt reaction of OTC was combined with the novel COF-DdBd nanocatalytic amplification to establish a simple and sensitive new SERS/RRS dual-mode method to detect the ultratrace OTC in a soil sample.

## 2. Experimental

### 2.1. Instruments and Reagents

Instruments: A model F-7000 fluorescence spectrophotometer (Hitachi Co., Tokyo, Japan) with the parameters of volt = 350 V, excited slit = emission slit = 5 nm; a DXR smart Raman spectrometer with a laser wavelength of 633 nm, a laser power of 3.0 mW, a slit of 25 µm and an acquisition time of 5 s (Thermo Fisher Scientific Co., Waltham, MA, USA); a model TU-1901 dual-beam UV−visible spectrophotometer (Beijing Puxi General Equipment Limited Co., Ltd., Beijing, China); a high-speed freezing centrifuge (Shanghai Lu Xiangyi Centrifuge Instrument Co., Ltd., Shanghai, China), an electric thermostatic drying oven (Shanghai Jing Hong Laboratory Instrument Co., Ltd., Shanghai, China); a Fourier transform infrared (FTIR) absorption spectrometer (Shanghai PerkinElmer Instruments Co., Ltd., Shanghai, China); an FEI Tecnai G2 F20 field-emission transmission electron microscope (Thermo Fisher Scientific Co., Ltd., Suzhou, China) with stem volt = 200 kV and camera constant 200; an X-ray diffractometer (Nippon Rigaku Co., Tokyo, Japan); Belsorp-max II automatic specific surface area and porosity distributor (Mack Company, Tokyo, Japan), sonic cleaner SK3300B (Shanghai Kedao Ultrasonic Instrument Co., Ltd., Shanghai, China); KQ3200DB CNC ultrasonic cleaner (Kunshan Ultrasonic Instrument Co., Ltd., Jiangsu, China); SXZ-4–10TC ceramic fiber muffle furnace (Shanghai Shibei Instrument Equipment Factory Co., Shanghai, China); and a model HH-S2 constant temperature water bath kettle (Jintan Dadi Automatic Instrument Co., Changzhou, China) were used.

Reagents: Dicyandiamine (Shanghai Macklin Biochemical Co., Ltd., Shanghai, China); p-benzaldehyde (Shanghai Macklin Biochemical Co., Ltd., Shanghai, China); dimethyl sulfoxide solution (DMSO) (Guangzhou Yuanchang Trade Co., Ltd., Guangzhou, China); ethanol (Chengdu Kelong Chemical Co., Ltd., Chengdu, China); and 0.1 nmol/L Apt of OTC with a base sequence of 5′-CGA CGC ACA GTC GCT GGT GCG TAC CTG GTT GCC GTT GTG T-3′ (Shanghai Shengong Bio-engineering Co., Shanghai, China) were used. A 1.00 g/L chloroauric acid (HAuCl_4_) solution was prepared by taking 250 μL of 4% chloroauric acid in a 10 mL centrifugal tube and diluting to the mark. An amount of 0.0496 g of OTC solid was put in a tube and diluted to 10 mL with water to obtain a 10.0 mmol/L OTC solution, then gradually diluted to 0.100 nmol/L with water. All reagents used were analytical grade, and the experimental water was secondary pure water.

### 2.2. Preparation of the Highly Catalytic DdBd Nanosol

Bonding the requirements of nanocatalytic analysis and referring to previous research [21], the preparation steps of DdBd nanosol were as follows. An amount of 0.378 g of dicyandiamide (4.5 × 10^−3^ mol) and 0.101 g of p-benzaldehyde (7.5 × 10^−2^ mol) were dissolved in 20 mL DMSO at a ratio of 6:1, and were completely dissolved via ultrasound, then the solution was transferred into the reaction kettle and heated for 3 h in the muffle furnace at 180 °C. It was then taken out and a uniform orange-yellow solution was obtained. The prepared bright yellow solution was different from the reference prepared [21]. The molar concentration of dicyandiamide represents the prepared DdBd concentration; that is, the DdBd concentration was 0.225 mol/L. We took 3 mL 0.225 mol/L DdBd and diluted the volume to 10 mL with water. A dialysis bag with a 3500 Da cut molecular mass was utilized to dialyze for 14 h, and the DdBd solution concentration was 0.0675 mol/L.

### 2.3. Procedure

Amounts of 100 μL 6.75 mmol/L DdBd solution, 100 μL 0.100 nmol/L Apt_OTC_ solution, and 0–1000 μL 0.100 nmol/L OTC solution were added to a 5 mL test tube with stopper and mixed evenly. Then, 120 μL 1.00 g/L HAuCl_4_ solution, 35 μL 10.0 mmol/L HCl solution, and 200 μL 15.0 g/L PEG600 solution were added, and diluted to 1.50 mL. The tube was put in an 80 °C water bath to heat for 45 min, then taken out and cooled with ice water. The RRS intensity was measured by a fluorescence spectrophotometer and the value at 370 nm (I_370nm_) was recorded. The sample without OTC solution was prepared as the blank (I_370nm_)_0_, and ∆I = I_370nm_ − (I_370nm_)_0_ was calculated. When 100 μL 10.0 μmol/L VBB solution was added, the SERS intensity at 1613 cm^−1^ (I_1613_) was measured; OTC was not added as the blank (I_1613_)_0_, and ∆I_1613_ = I_1613_ − (I_1613_)_0_ was calculated.

## 3. Results and Discussion

### 3.1. Analytical Principles

**Figure 1:**
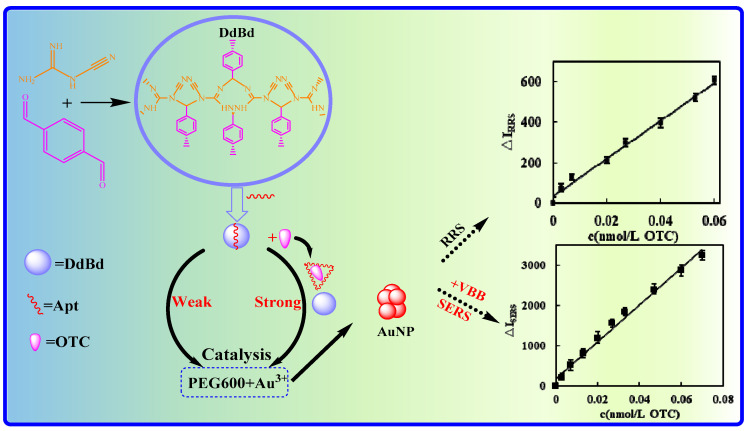
Apt-mediated DdBd nanocatalysis-gold nanosol plasma RRS/SERS dual scattering detection of OTC.

Three COFs were prepared by reacting cyanamide, dicyandiamide, and melamine with p-benzaldehyde. The experiment found that the reaction of HAuCl_4_-PEG600 to form gold nanoparticles (AuNPs) is very slow; in this gold nanoreaction system the COF could catalyze the reaction to form more AuNPs, and the generated AuNPs have RRS and SERS signals. Using the slope procedure, COF (DdBd) prepared by dicyandiamide-p-benzaldehyde showed the best catalytic effect and was selected for use. Considering the fact that both Apt_OTC_ and DdBd have functional groups and π-π overlapping hydrophobic groups, DdBd will be wrapped by Apt_OTC_ through hydrogen bonds, hydrophobic interactions, and intermolecular force interactions, causing its catalytic activity to be inhibited. When the OTC target was added, it could specifically bind to the Apt to form a stable complex and release free DdBd, thereby restoring the catalytic activity of DdBd and gradually increasing the content of AuNPs in the system. As a result, due to the increase in AuNPs, the RRS/SERS signal also gradually increased. Within a certain concentration of OTC range, the signal intensity of RRS/SERS is proportional to the concentration of added OTC. On this basis, a sensitive SERS/RRS double-scattering spectroscopy method can be established to determine OTC (Figure 1). Although the SERS/RRS peak area also has a linear relationship with the OTC concentration, it is not convenient and fast for peak height measurement. Therefore, its intensity is chosen to be expressed in the following text.

### 3.2. Characterization of the Nanomaterials

**Figure A1:**
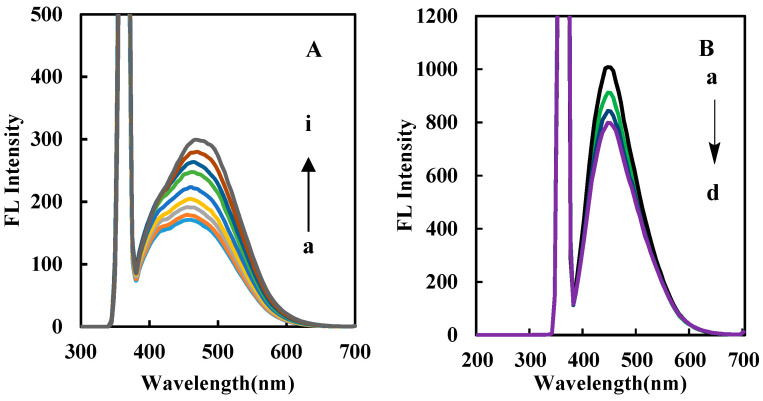

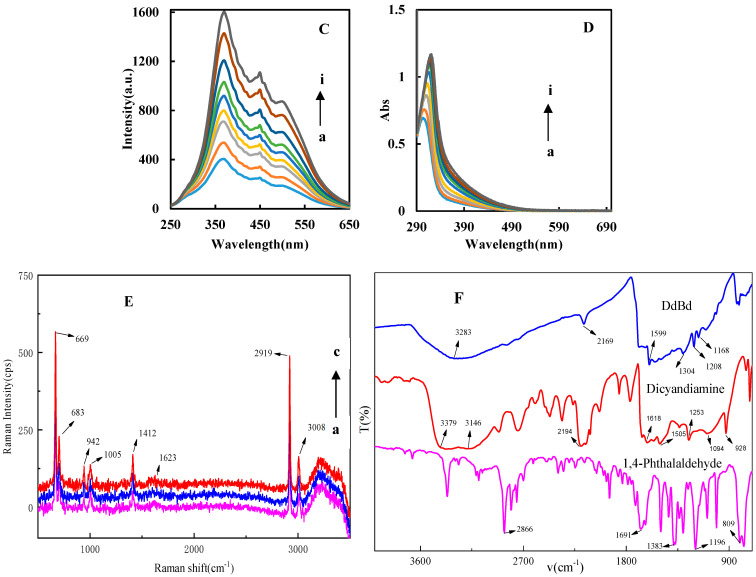
Molecular spectra of DdBd. (**A**) Fluorescence spectrum of DdBd (a–i: 10.7, 11.8, 12.5, 13.2, 15.0, 17.3, 18.8, 20.5, 22.5 mmol/L DdBd, with volt = 500 V, excited slit = emission slit = 5 nm). (**B**) Fluorescence spectrum of 0.680 mmol/L DdBd+Apt_OTC_ (a–d: 0, 20.0, 40.0, 50.0 nmol/L Apt_OTC_, with volt = 500 V, excited slit = emission slit = 10 nm). (**C**) RRS spectrum of DdBd (a–i: 2.25, 3.21, 4.09, 4.89, 5.63, 6.30, 6.92, 7.50, 8.04 mmol/L DdBd). (**D**) Absorption spectrum of DdBd (a–i: 2.25, 2.74, 3.66, 4.29, 5.08, 6.46, 7.07, 7.50, 8.04 mmol/L DdBd). (**E**) Normal Raman spectrum (a–c: 14.5, 17.6, 22.5 mmol/L DdBd). (**F**) FTIR spectrum of dicyandiamide, p-benzaldehyde, and DdBd.

**Figure A2:**
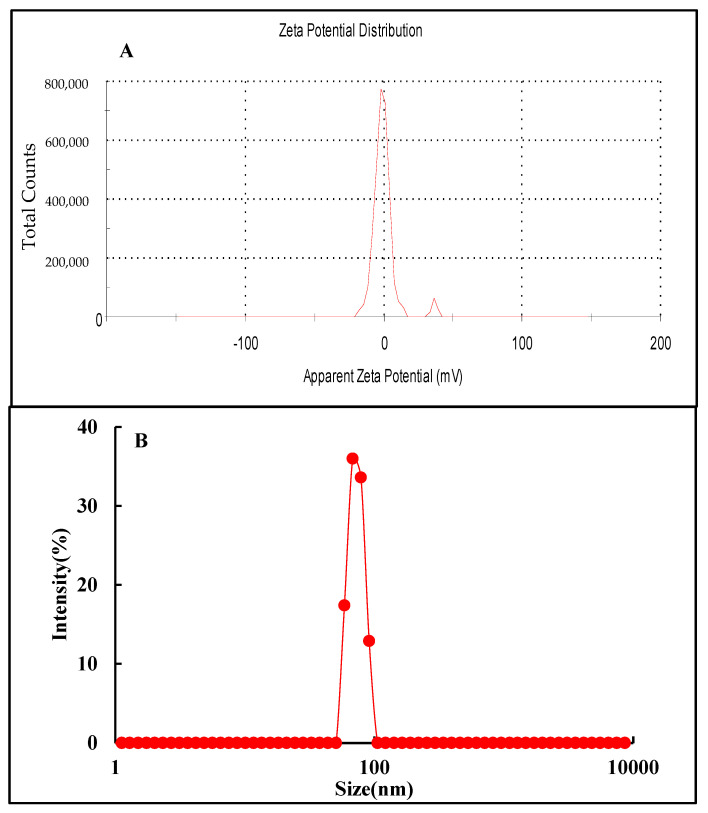
Apparent zeta potential and particle size distribution of DdBd. (**A**) Apparent zeta potential of DdBd. (**B**) DdBd particle size distribution.

An ordered π-π structure exists in COF materials, and the luminescence characteristics of the π structure can be utilized as a fluorescent molecular probe to develop an analysis method. In the COF materials, however, the aggregation of the π-π overlayer may also cause fluorescence quenching. DdBd has a fluorescence peak at 460 nm under the excitation wavelength of 360 nm (Figure A1A), and the fluorescence gradually increases with the increase in DdBd concentration. It has been found that the interaction between aptamers and nanomaterials with fluorescent properties will lead to its fluorescence decrease [22]. Moreover, due to the fluorescence characteristics of DdBd, it was used as a direct fluorescence probe to examine the interaction between the COF and the aptamer. The result (Figure A1B) shows that the fluorescence decreased with the aptamer increasing from 0 to 50 nmol/L aptamer. This demonstrates that when DdBd is wrapped by Apt, the excited light cannot directly hit the surface of DdBd, making it difficult for it to enter the excited state. Without the transition of electrons, energy will not be released to produce fluorescence, so the fluorescence of DdBd will be weakened. The RRS spectrum (Figure A1C) reveals that DdBd produces two intense RRS peaks at 360 nm and 450 nm. With the increase in DdBd concentration, the two peak RRS signals also increase due to the nanoparticles increasing, and the peak at 450 nm is more sensitive. The absorption spectrum of DdBd (Figure A1D) has an absorbing peak at 310 nm. The hyperchromic response of the absorption peak also increases with the DdBd concentration increase, and the higher the concentration, the greater the degree of conjugation of the nanoparticles, which cause the ultraviolet absorption peak red-shift. The characteristic peaks of Raman at 1005 cm^−1^ and 1623 cm^−1^ (Figure A1E) are attributable to a benzene ring stretching vibration and C=C bond stretching vibration, and because dicyandiamide does not have the same ring structure as melamine, the peak at 1623 cm^−1^ is very weak. The two peaks at 669 cm^−1^ and 683 cm^−1^ can be ascribed to the N-H bond deformation vibration, and the peak at 1412 cm^−1^ is attributed to the C-N bond tensile vibration. The peaks at 2919 cm^−1^ and 3008 cm^−1^ can be ascribed to the N-H bond stretching vibration, which is in accordance with the characterization of infrared results. According to the preparation method of the DdBd materials, the solution was obtained and placed in a 50 mL centrifugal tube and centrifuged with a refrigerated centrifuge. The supernatant liquid was discarded and 20–30 mL water was added for washing; this was repeated 3–4 times, and after adding 20 mL water the resulting precipitate was ultrasonically dispersed. Finally, dried solid DdBd was obtained after oven drying for 1–2 h. The precursors dicyandiamine and p-benzaldehyde were used to record the FTIR (Figure A1F). DdBd exhibits broad and large frequency bands at 3283 cm^−1^ and a peak at 2169 cm^−1^, which are ascribed to dicyandiamide and which indicate the N-H bond stretching vibration and symmetrical stretching vibration of -NH_2_ bonds. Both of them have C≡N bond extra tensile vibrations at 2194 cm^−1^ and 2169 cm^−1^, and N-H deformation vibrations at 1253 cm^−1^ and 1208 cm^−1^. Of course, dicyandiamide also has C=N bonds with obvious tensile vibrations at 1618 cm^−1^ and 1505 cm^−1^, and DdBd also has this key at 1599 cm^−1^. The peak relevant to the C=O bond stretching vibration that disappeared at 1691 cm^−1^ indicates that the reaction of aldehyde occurred and the triazine unit assimilated into the structure successfully. The peaks of dicyandiamine at 1094 cm^−1^ and DdBd at 1168 cm^−1^ also show the presence of the secondary amine C-N tensile vibration. At the same time, in dicyandiamide, the C-N-C bond symmetric stretching vibration existing at 928 cm^−1^ disappears in DdBd with the change in the ring structure of COF. Zeta potential is a measure of the strength of mutual repulsion or attraction between particles. Under neutral conditions, the zeta potential of DdBd is negative. It can be seen from Figure A2A that the charge distribution of DdBd is −1.29 Mv, and the ζ value of the Zeta potential is between 0 and ±5. This proves that the repulsive force between particles is small, and particles are prone to aggregation. As shown in Figure A2B, the particle size distribution of DdBd was measured with a particle size analyzer, and this shows that the mean granule size of DdBd was approximately 68 nm.

**Figure 2:**
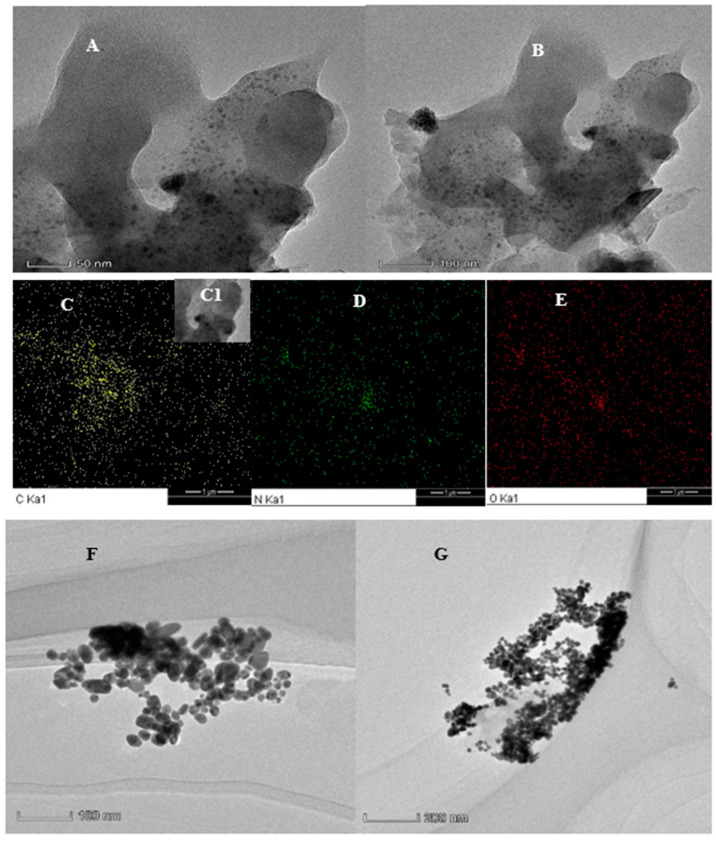
(**A**,**B**) TEM image of DdBd. (**C**–**E**) The mapping image of DdBd. (**C1**) TEM image of the element mapping part. (**F**) TEM image of 80.0 mg/L HAuCl_4_ + 0.230 mmol/L HCl + 149 g/L PEG + 0.300 mmol/L DdBd + 6.70 pmol/L Apt_OTC_ + 0.625 μmol/L VBB. (**G**) TEM image of F + 4.00 × 10^−2^ nmol/L OTC.

**Figure A3:**
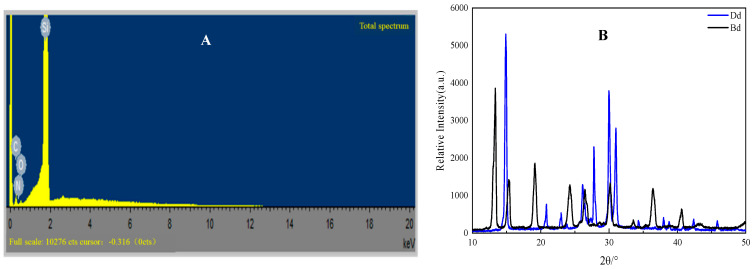

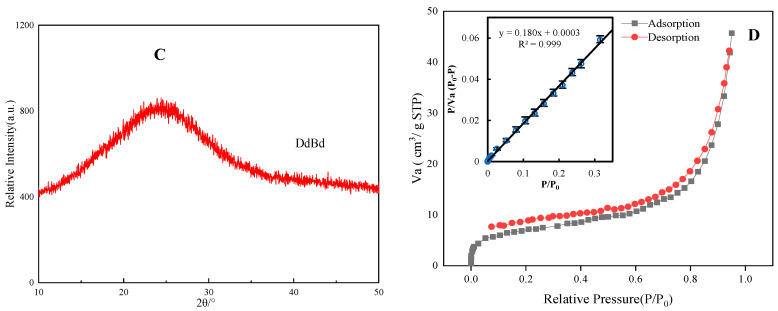
XRD, adsorption/desorption isotherms and energy spectrum of DdBd. (**A**) DdBd energy spectrum. (**B**) XRD of Dd and Bd. (**C**) XRD of DdBd. (**D**) N_2_ adsorption/desorption isotherms of DdBd (where P is the measured air pressure, P_0_ is the saturated vapor pressure of nitrogen at liquid nitrogen temperature, P/P_0_ is the partial pressure of N_2_).

Transmission electron microscope (TEM) images of DdBd and the analysis system were recorded. As shown in Figure 2A,B, the degree of aggregation of particles in DdBd is small, and the dispersion is relatively uniform, which is in keeping with the average particles in the particle size analyzer. The particle diameter of a single particle is approximately 70 nm, but from Figure 2B, we can see that smaller particles are spreading around, and the diameter of a single particle is approximately 40 nm. In the element distribution diagram, it can be clearly seen that the C, N, and O elements are distributed in various places, and the distribution of elements in the concentrated area of DdBd is more dense (Figure 2C,E). The energy spectra show that the DdBd particles mainly contain C, N, and O elements (Figure A3A). So as to check the degree of crystallinity of the prepared DdBd, the sample and two of its precursors were tested via powder X-ray diffraction. As shown in Figure A3B,C, the two precursors of DdBd have good crystallinity, and the DdBd also has a definite degree of crystallinity, which can be confirmed when the Bragg angle is 25° and the DdBd has a wide diffraction peak. This is in accordance with the TEM image. The sample DdBd was degassed at 150 °C for 8 h, then tested with the BELSORP-MAX II automatic specific surface area and porosity distribution instrument under an N_2_ atmosphere. During the test, we ensured that the sample tube was completely immersed in liquid nitrogen. The specific surface area of the sample was calculated by BET: S_BET_ = 24.2 m^2^/g. This shows that the prepared DdBd has a good specific surface area, which also provides a good site for aptamer adsorption (Figure A3D). The AuNP produced in the analysis system without OTC is quasi-spherical with a mean size of 30 nm; after addition of OTC, the catalytic indicator nanoreaction enhanced to form more AuNPs with a size of 20 nm (Figure 2F,G).

### 3.3. RRS and SERS Spectra of the Catalytic Analysis System

**Figure 3:**
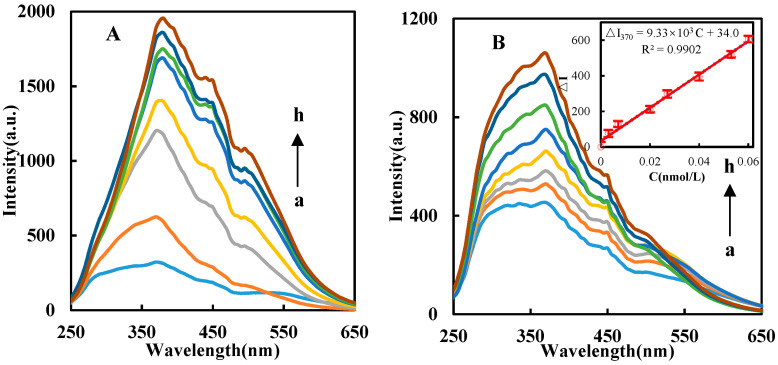

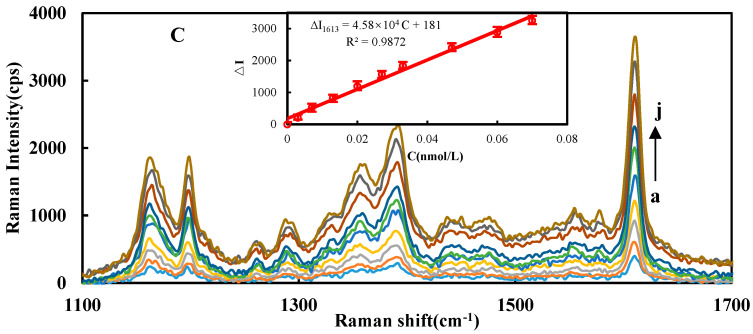
RRS and SERS spectra of the catalytic analysis system. (**A**) RRS spectra of catalytic system: 53.0 mg/L HAuCl_4_ + 0.300 mmol/L HCl + 2.00 g/L PEG600+ DdBd (a–h: 0, 0.225, 0.450, 0.675, 0.900, 1.13, 1.35, 1.58 mmol/L DdBd). (**B**) RRS spectra of catalytic analysis system: 0.080 g/L HAuCl_4_ + 0.230 mmol/L HCl + 2.00 g/L PEG600 + 0.450 mmol/L DdBd + 6.70 pmol/L Apt + OTC (a–h: 0, 3.00 × 10^−3^, 6.70 × 10^−3^, 2.00 × 10^−2^, 2.70 × 10^−2^, 4.00 × 10^−2^, 5.30 × 10^−2^, 6.00 × 10^−2^ nmol/L OTC). Inset shows a linear relationship between the RRS intensity(△I_370_) with the OTC concentration. (**C**) SERS spectra of catalytic analysis system: 0.080 g/L HAuCl_4_ + 0.230 mmol/L HCl + 2.00 g/L PEG600 + 0.450 mmol/L DdBd + 6.70 pmol/L Apt_OTC_+0.625 μmol/L VBB+OTC (a–j: 0, 3.00 × 10^−3^, 6.70 × 10^−3^, 1.30 × 10^−2^, 2.00 × 10^−2^, 2.70 × 10^−2^, 3.30 × 10^−2^, 4.70 × 10^−2^, 6.00 × 10^−2^, 7.00 × 10^−2^ nmol/L OTC). Inset shows a linear relationship between the SERS intensity(△I_1613_) with the OTC concentration.

**Figure A4:**
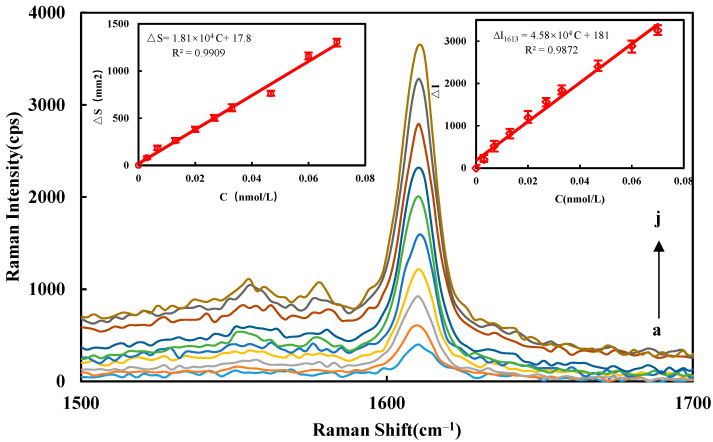
The relationship between SERS intensity and area with OTC concentration. The DdBd-PEG600-HCl-HAuCl4-Apt_OTC_-OTC-VBB catalytic analysis system: 0.080 g/L HAuCl4 + 0.230 mmol/L HCl + 2.00 g/L PEG600 + 0.450 mmol/L DdBd + 6.70 pmol/L AptOTC + 0.625 μmol/L VBB+OTC (a–j: 0, 3.00 × 10^−3^, 6.70 × 10^−3^, 1.30 × 10^−2^, 2.00 × 10^−2^, 2.70 × 10^−2^, 3.30 × 10^−2^, 4.70 × 10^−2^, 6.00 × 10^−2^, 7.00 × 10^−2^ nmol/L OTC).

**Figure A5:**
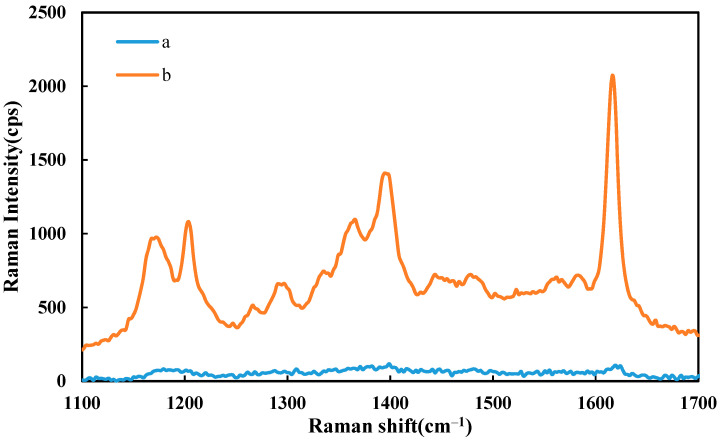
Normal Raman and SERS spectra of the VBB. (**a**) Normal Raman spectra of 0.1 mol/L VBB solution. (**b**) SRES spectra of catalysis system: 0.080 g/L HAuCl_4_ + 0.230 mmol/L HCl + 2.00 g/L PEG600 + 0.450 mmol/L DdBd + 0.625 μmol/L VBB.

Highly sensitive SERS and simple RRS are a kind of inelastic and elastic scattering, respectively, and both have developed rapidly with nanoparticle preparation technology. The dimode molecular spectra were chosen to study the COF catalytic indicator reaction. The AuNP indicator reaction of PEG600-HAuCl_4_ is extremely slow, and the RRS signal is very weak due to fewer AuNPs. However, the prepared DdBd has a strong catalytic effect on this nanoreaction. The system has two RRS peaks at 370 nm and 520 nm (Figure 3A), and the largest peak is at 370 nm. Therefore, this spectral peak was selected for the RRS analysis. The catalytic effect of COF depends not merely on its chemical properties, but also on its size. A large size of COF displays weak catalysis, which is similar to precious nanoparticles. With the amount of DdBd increased, the catalytic effect, the produced AuNPs, and the RRS signal intensity ΔI_370nm_ gradually increased. For the DdBd-PEG600-HAuCl_4_-Apt_OTC_-OTC nanocatalytic system, via electrostatic adsorption Apt can adsorb on the surface of DdBd to restrain its catalytic action. After adding the OTC target, Apt combines with the target and releases DdBd, restoring the DdBd catalytic activity. The ∆I_370nm_ shows a great linear relation with OTC concentration (Figure 3B), so we selected this peak for the determination of OTC. In general, large particles produce strong RRS. Owing to the formation of large AuNPs in the blank, the RRS blank was high. SERS is a highly sensitive molecular spectral technique. We used it to research the aptamer-mediated catalytic analysis system. However, there was no SERS signal when SERS detection is performed. Thankfully, in the presence of the SERS probe molecule Victoria blue B (VBB), the system showed an intense SERS peak at 1613 cm^−1^, belonging to C=N and C=C bond bending stretching. The SERS peak at 1164 cm^−1^ is attributed to CH_2_ bond bending vibration. The peak values at 1360 cm^−1^ and 1393 cm^−1^ are attributed to the symmetric bending vibration of the CH_2_ bond and bending vibration of the CH bond, respectively. The peak at 1199 cm^−1^ is attributed to symmetric tensile vibration of the NH_2_ bond (Figure 3C). The signal strength ΔI_1613cm_^−1^ is positively proportional to the concentration of OTC, with a detection limit of 9.0 × 10^−4^ nmol/L. In addition, the linear relationship between the area and intensity of SERS with OTC concentration was compared (Figure A4), and it was found that using SERS intensity to express OTC concentration showed a higher slope and higher sensitivity. Its determination of sensitivity is higher than with the RRS method. Additionally, due to the large AuNPs and the weaker SERS activity, the blank value is lower than the RRS. Compared with RRS, this is the advantage of SERS. Therefore, the SERS of the PEG600 system was chosen to detect OTC. In addition, according to the calculation formula of enhancement factor (EF) [23,24], EF = (I_SERS_/C_SERS_)/(I_RS_/C_RS_), the Raman spectra of the VBB solution with AuNPs and VBB solution were recorded (Figure A5); I_SERS_ = 1836, C_VBB_ = 6.25 × 10^−7^ M, when VBB solution C_RS_ = 0.1 M and its intensity I_RS_ = 80.04, thus the EF is 3.67 × 10^6^. Generally, the EF results were 10^3^~10^14^ [25]; EF in this study was 10^6^, indicating that the newly prepared AuNPs showed good performance as a SERS substrate. Compared with the reported EF of rhodamine 6G (R6G) and crystal violet (CV) common molecular probes, the EF of R6G and CV in the study of Wang et al. were 6.12 × 10^7^ and 3.02 × 10^5^, respectively [26]. Although the EF_VBB_ molecular probe in this study was not as good as R6G, there exists fluorescence interference. Compared with the CV, the VBB shows a better Raman enhancement effect.

### 3.4. Catalytic Enhancement Mechanism of COF

**Figure 4:**
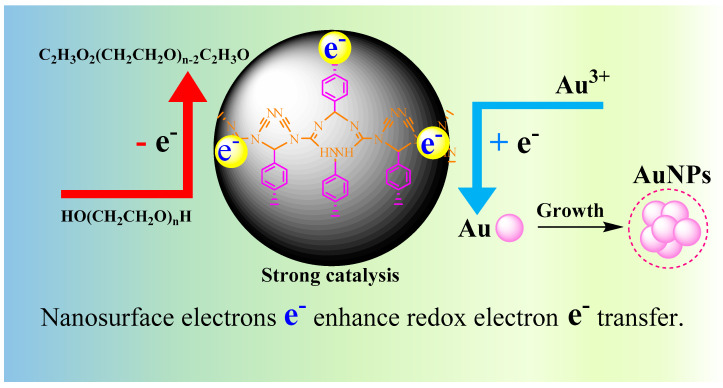
DdBd catalytic enhancement mechanism.

Under the experimental conditions, DdBd can catalyze the reduction of HAuCl_4_ by PEG600 to form gold nanoparticles. Within a certain concentration range, with the concentration of the catalyst DdBd increases, the catalytic capacity increases, and the generated gold nanoparticles increase. At the same time, the RRS and SERS effects of the system increase and show a certain linearity with the concentration of DdBd. Here we still compare the catalytic effects of DdBd by changing the reducing agent (Table A1). The results show that the PEG600 exhibits the biggest slope and it was chosen for use. Dicyandiamide does not have the same ring structure as melamine, but it has C≡N. Therefore, the DdBd synthesized by the condensation reaction of dicyandiamine and p-benzaldehyde has a porous organic structure with a skeleton, and it is easier to obtain the structure of 3D. By combining the catalytic active center embedded in the material skeleton, it has better catalytic performance. Combined with the characteristics of low density, large specific surface area and good chemical stability of COF, this new COF may bring new vitality to the research. In short, the surface electrons on DdBd can accelerate the redox electron-transfer between Au^3+^ and PEG600 to speed up the AuNP reaction (Figure 4).

### 3.5. Optimal Conditions

**Figure A6:**
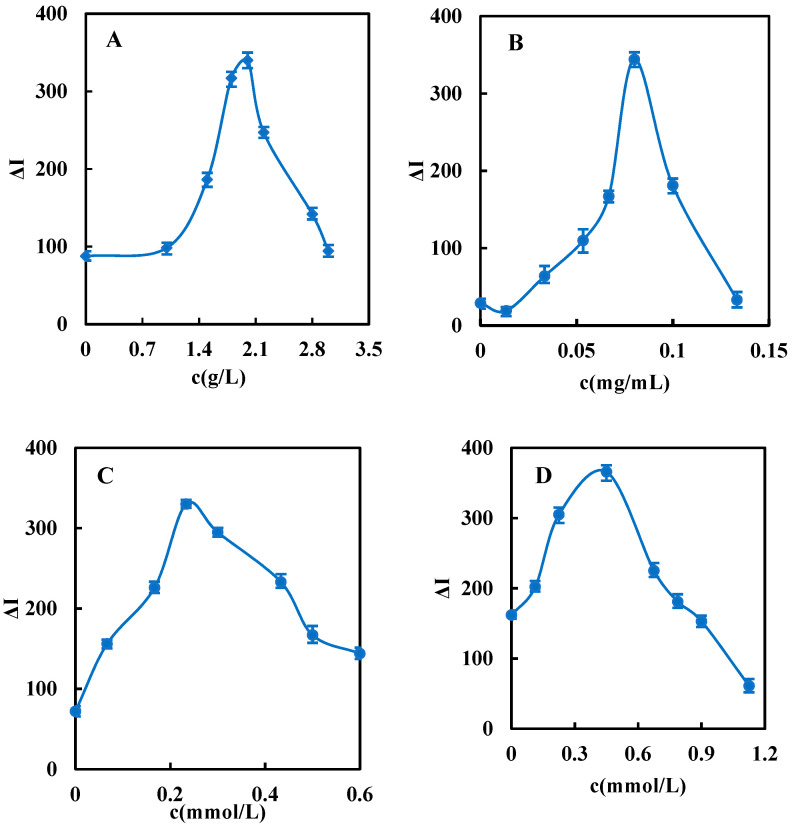

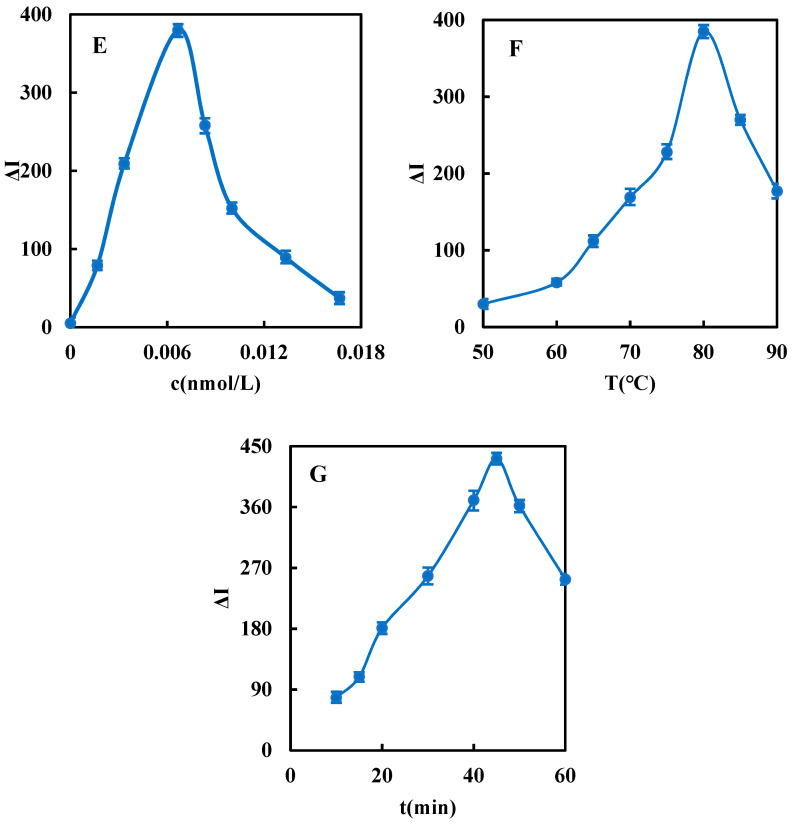
Optimization of analysis conditions. (**A**) Effect of PEG600 concentration. (**B**) Effect of HAuCl_4_ concentration. (**C**) Effect of HCl concentration. (**D**) Effect of DdBd concentration. (**E**) Effect of Apt_OTC_ concentration. (**F**) Effect of reaction temperature. (**G**) Effect of reaction time.

The effects of PEG600, HAuCl_4_, HCl, Apt_OTC_, the concentration of DdBd, the temperatures of reaction and time on the scattering signals of the system were studied separately (Figure A6). When 2.00 g/L PEG600, 80.0 mg/L HAuCl_4_, 0.230 mmol/L HCl, 0.450 mmol/L DdBd, 6.70 pmol/L Apt_OTC_, 0.040 nmol/L OTC, and temperature of 80 °C for 45 min were selected, the signal was strongest. In addition, the 0.625 μmol/L VBB concentration was chosen for use.

### 3.6. Working Curve

Under the chosen conditions, different concentrations of OTC, and the according RRS, SERS signal values were used to draw the working curves to obtain their analytical features (Table 1). The linear range of RRS (LR) is 3.00 × 10^−3^–6.00 × 10^−2^ nmol/L, the linear equation is ΔI_370nm_ = 9.33 × 10^3^ C + 34.0, the coefficient is 0.9902, and the limit of detection (DL) is 1.1 × 10^−3^ nmol/L. The SERS linear range is 3.00 × 10^−3^–7.00 × 10^−2^ nmol/L, the linear equation is ΔI_1613_ = 4.58 × 10^4^ C + 181, the coefficient is 0.9872, and the detection limit is 9.0 × 10^−4^ nmol/L. The RRS and SERS working curves are shown in Figure 3. Through comparison, the RRS is simpler than SERS due to its determination and does not require SERS molecular probes. However, it can be seen that the SERS analysis method is not only highly sensitive, but also has low blank value and good reproducibility; it has low blank value due to large AuNPs with low SERS activity. It is more practical than the RRS analysis method. Therefore, the SERS method was selected to detect the OTC in the sample. Although the use of COF and SERS analysis methods has been reported to determine the analyte, it can be seen from Table A2 that the OTC detection methods reported in recent years are not enough [27,28,29,30,31], the sensitivity is not high, and the quantitative analysis method of SERS is not used to detect OTC in soil samples. This method provides a new method to detect OTC in soil samples.

### 3.7. Influence of Coexisting Substances

On the basis of the experimental method, the influence of coexisting substances in the detection of 1.30 × 10^−2^ nmol/L OTC by RRS and SERS was researched. The outcomes of the experiment indicate that within a relative error range not exceeding ±10%, in the RRS spectrum analysis, 1000 times Na^+^, Co^2+^, Ca^2+^, SO_3_^2−^, PO_4_^3−^, NO_2_^−^, CH_3_COO^−^, lysine (Lys), histidine (His), and tryptophan (Trp); 500 times Fe^3+^, SO_4_^2−^, HCO_3_^−^, HPO_4_^2−^, CO_3_^2−^, and alanine (Ala); 100 times Mg^2+^, P_2_O_7_^4−^, glycine (Gly), and threonine (Thr); 50 times K^+^; 10 times H_2_PO_4_^−^ and penicillin sodium; and 5 times tetracycline did not interfere with the RRS measurement of OTC (Table A3). In the SERS spectral analysis, 1000 times Na^+^, Co^2+^, Mg^2+^, Fe^3+^, SO_3_^2−^, PO_4_^3−^, NO_2_^−^, CH_3_COO^−^, H_2_PO_4_^−^, threonine (Thr), Lys, and Ala; 500 times Ca^2+^, HPO_4_^2−^, P_2_O_7_^4−^, and His; 100 times K^+^, HCO_3_^−^, CO_3_^2−^, and Trp; 50 times Al^3+^; 10 times Zn^2+^, SO_4_^2−^, and Gly; and 5 times ofloxacin and tetracycline did not interfere with OTC SERS determination (Table A4). This shows that the selectivity of these two spectral analysis methods is relatively good.

### 3.8. Sample Analysis

Four soil samples were collected, dried, smashed, and ground. We weighed 0.200 g of each soil sample into a 50 mL centrifuge tube, added water to increase the volume to 30 mL, used an ultrasonic cleaner to sonicate the mixture for 36 h, and then centrifuged for 10 min at 10,000 r/min. The supernatant liquid was used to detect the OTC content after passing through a 0.2 μm filter membrane to remove the residue. The blank without sample as the blank solution was disposed of in the same way, and was detected depending on the experimental method. The final measurement results were on the basis of the mean of 5 measurements. The measurement results are shown in Table A5. The relative standard deviation (RSD) is 1.35~4.78%, and the sample recovery is 94.3~104.9%. This shows that the SERS method is reliable and accurate.

## 4. Discussion

It was found that redox electron transfer between Au^3+^ and PEG600 is very slow, and AuNPs are rarely formed. For the prepared material DdBd, its surface electrons can accelerate the redox electron transfer between Au^3+^ and PEG600, which speeds up the formation of AuNPs. When DdBd was wrapped via the Apt_OTC_, the synergy between nanosurface electrons of DdBd and the redox electrons was destroyed, the nanoreaction between Au^3+^ and PEG600 was inhibited, so the generated AuNPs decreased, and the SERS/RRS signal changes correlated with the change in AuNP content. Upon addition of the target molecule OTC, it specially bound Apt_OTC_ to release DdBd, which sped up the transfer of redox electrons, and the SERS/RRS signal increased with the OTC increasing. Based on this theory, this research has established a new SERS/RRS method for OTC detection.

The COF exhibits strong catalysis of the Au(III)-PEG reaction, which can amplify the analytical signal. This is a foundation for developing a highly sensitive SERS/RRS method. Secondly, the interaction of COF and Apt should be appropriate. The strong interaction between them leads to the difficulty in Apt desorption, and the assay method cannot be developed. Too weak an interaction caused a high blank. Finally, the specificity of the Apt is high. After reacting with the target molecule, the Apt can be desorbed from the COF surface so as to obtain a highly sensitive and selective method.

## 5. Conclusions

According to the solvothermal reaction of dicyandiamide and p-benzaldehyde, DdBd nanosols with high catalysis and stability were prepared and characterized via TEM, EDS, infrared spectroscopy, and other techniques in detail. It was found that DdBd has an intense catalytic activity on the new PEG600-HAuCl_4_ nanoreaction, and the generated AuNPs show intense SERS signals in the presence of VBB molecular probes. Via combining the Apt reaction with the DdBd nanocatalytic amplification reaction, a new SERS quantitative analysis method to detect OTC was developed. It has advantages of high sensitivity, selectivity, accuracy, and low blank value. In addition, RRS can also be used to detect OTC without adding molecular probes. This work can provide new ideas for the design of the Apt-controlling COF catalytic AuNP reaction SERS system, and this system can be used to detect residual antibiotic substances in the environment and eliminate the potential harm of these substances to the natural environment and human body.

## Figures and Tables

**Table 1:** RRS/SERS analysis features of HAuCl_4_-glycol (GC)/PEG-Apt-DdBd-OTC system.

Reductant	Linear Range (×10^−3^ nmol/L)	Linear Equation	Coefficient	DL (×10^−3^ nmol/L)
EG	13.0–53.0 6.70–33.0	ΔI_RRS_ = 2.54 × 10^3^ C_OTC_ + 11.9 I_SERS_ = 1.44 × 10^4^ C_OTC_ − 92.5	0.9853 0.9797	8.7 7.2
PEG200	6.70–46.7 6.70–53.0	ΔI_RRS_ = 7.30 × 10^3^ C_OTC_ + 13.9 I_SERS_ = 2.50 × 10^4^ C_OTC_ + 59.4	0.9721 0.9932	3.3 2.2
PEG400	13.0–60.0 6.70–60.0	ΔI_RRS_ = 2.99 × 10^3^ C_OTC_ − 8.10 I_SERS_ = 4.00 × 10^4^ C_OTC_ + 308	0.9715 0.9292	5.0 5.0
PEG600	3.00–60.0 3.00–70.0	ΔI_RRS_ = 9.33 × 10^3^ C_OTC_ + 33.9 I_SERS_ = 4.58 × 10^4^ C_OTC_ + 181	0.9902 0.9872	1.1 0.9
PEG6000	6.70–57.0 3.30–33.0	ΔI_RRS_ = 7.76 × 10^3^ C_OTC_ + 0.400 I_SERS_ = 2.93 × 10^4^ C_OTC_ − 0.800	0.9748 0.9899	2.9 2.6
PEG20000	6.70–50.0 6.70–43.0	I_RRS_ = 6.19 × 10^3^ C_OTC_ + 18.2 I_SERS_ = 171 × 10^4^ C_OTC_ − 13.9	0.9905 0.9801	3.2 3.8

## Data Availability

Not applicable.

## Appendix A

**Table A1:** Comparison of DdBd nanocatalysis characteristics of glycol/PEG systems.

Nano Catalytic System	Linear Range (nmol/L)	Linear Equation	Coefficient
lycol	0.450–4.50	ΔI_370nm_ = 1.73 × 10^3^ C − 67.4	0.9750
PEG200	0.225–1.575	ΔI_370nm_ = 5.54 × 10^3^ C + 402.3	0.9208
PEG400	0.450–3.15	ΔI_370nm_ = 3.26 × 10^3^ C + 383	0.9603
PEG600	0.225–1.58	ΔI_370nm_ = 6.67 × 10^3^ C + 223	0.9952
PEG6000	0.225–1.58	ΔI_370nm_ = 2.79 × 10^3^ C + 57.9	0.9590
PEG20000	0.225–1.58	ΔI_370nm_ = 5.11 × 10^3^ C + 231.3	0.9301

**Table A2:** Comparison of reported OTC analysis methods.

Method	Principle	Linear Range	DL	Ref.
Fluorescence	The covalent attachment of DNA probes and MOF nanosheets enhances the affinity between the nanomaterials and DNA, and the interaction with OTC leads to a decrease in fluorescence.	0.50–5.00 μg/L	0.40 μg/L	[27]
Apt electrochemical sensor	A portable aptamer sensor was constructed to detect oxytetracycline by using gold nanoparticles/carboxylated multi-walled carbon nanotubes @thionine-linked aptamer complementary chains as signal markers.	10^−13^–10^−5^ g/mL	3.1 × 10^−14^ g/mL	[28]
Apt electrochemical sensor	The Ce-MOF@COF hybrid product shows high bioaffinity for OTC targeting aptamers, which further increases the detection effect of OTC detection.	0.1–0.5 ng/mL	17.4 fg/mL	[29]
Ap light sensor	A LRET biosensor was developed, coupling the OTC aptamer to the photonanoparticle for OTC measurement.	0.1–10 ng/ml	0.054 ng/ml	[30]
SERS	A nano-biosensor based on AuNPs was established, with stem-loop DNA modification as the SERS active substrate for OTC detection.	4.60 × 10^−2^–4.6 × 10^2^ fg/mL	4.35 × 10^−3^ fg/mL	[31]
SERS/RRS	According to a COF-catalyzed PEG600-HAuCl4-HCl reaction to generate AuNPs, SERS and RRS signals were generated, and combined with aptamers at the same time; a new SERS/RRS coupled dual-mode OTC detection method was developed.	3.00 × 10^−3^–7.00 × 10^−2^ nmol/L	9.0 × 10^−4^ nmol/L	This Method

**Table A3:** Influence of coexistent substances (RRS).

Coexisting Substances	Times	Relative Error (%)	Coexisting Substances	Times	Relative Error (%)
Na^+^	1000	−2.4	HCO_3_^−^	500	2.4
Co^2+^	1000	4.5	CH_3_COO^−^	1000	−2.1
Zn^2+^	500	3.3	HPO_4_^2^^−^	500	6.1
K^+^	50	4.4	H_2_PO_4_^−^	10	−5.3
Ca^2+^	1000	3.5	P_2_O_7_^4^^−^	100	9.6
Mg^2+^	100	−4.3	CO_3_^2^^−^	500	−7.5
Al^3+^	1000	5.2	NAD	100	−5.3
Fe^3+^	500	3.1	GTP	1000	−7.2
SO_3_^2^^−^	1000	−6.4	CTP	1000	−6.3
SO_4_^2^^−^	500	−4.5	UTP	1000	−4.1
PO_4_^3^^−^	1000	−5.1	ADP	500	−2.3
NO_2_^−^	1000	−3.1	AMP	100	6.8
penicillin sodium	10	5.4	tetracycline	5	8.6

**Table A4:** Influence of coexistent substances (SERS).

Coexisting Substances	Times	Relative Error (%)	Coexisting Substances	Times	Relative Error (%)
Na^+^	1000	−5.9	HCO_3_^−^	100	−1.8
Co^2+^	1000	5.9	CH_3_COO^−^	1000	−7.1
Zn^2+^	10	−3.5	HPO_4_^2^^−^	500	5.4
K^+^	100	−5.3	H_2_PO_4_^−^	1000	−0.6
Ca^2+^	500	−3.4	P_2_O_7_^4^^−^	500	−6.1
Mg^2+^	1000	−6.7	CO_3_^2^^−^	100	−3.5
Al^3+^	50	2.8	Thr	1000	−4.7
Fe^3+^	1000	4.2	Lys	1000	1.1
SO_3_^2^^−^	1000	2.8	His	500	6.1
SO_4_^2^^−^	10	−9.5	Trp	100	2.8
PO_4_^3^^−^	1000	1.7	Ala	1000	4.3
NO_2_^−^	1000	−6.9	Gly	10	−5.7
ofloxacin	5	6.7	tetracycline	5	7.1

**Table A5:** The results of OTC sample determination.

Sample	Single Value (×10^−3^ nmol/L)	Average (×10^−3^ nmol/L)	Added (×10^−3^ nmol/L)	Found (×10^−3^ nmol/L)	Recovery (%)	RSD (%)	Content (μg/kg)	AAS (μg/kg)
1	5.45, 5.36, 5.73, 5.01, 5.36	5.38	6.70	11.88	96.3	4.78	2.04	
			13.0	18.79	107.6			2.20
			20.0	25.52	102.6			
2	7.04, 6.73, 6.96 7.23, 7.18	7.03	6.70	13.42	95.6	2.82	2.67	
			13.0	20.39	105.1			2.44
			20.0	27.10	101.1			
3	8.49, 8.47, 8.34 8.61, 8.73	8.53	6.70	15.16	99.2	1.73	3.24	
			13.0	21.42	98.7			3.46
			20.0	28.17	95.8			
4	7.82, 7.83, 7.65 7.93, 7.74	7.79	6.70	14.05	94.3	1.35	2.96	
			13.0	20.93	101.8			3.12
			20.0	28.17	104.9			
